# Evolving therapeutic landscape in follicular lymphoma:* a look at emerging and investigational therapies*

**DOI:** 10.1186/s13045-021-01113-2

**Published:** 2021-06-30

**Authors:** Walter Hanel, Narendranath Epperla

**Affiliations:** 1 Division of Hematology, Department of Medicine, The James Cancer Hospital and Solove Research Institute, The Ohio State University, 460 W 10th Ave, Columbus, OH 43210 USA; 2 The Ohio State University Comprehensive Cancer Center, 1110E Lincoln Tower, 1800 Cannon Drive, Columbus, OH 43210 USA

**Keywords:** Follicular lymphoma, PI3Ki, EZH2, CART, BiTe

## Abstract

Follicular Lymphoma (FL) is the most common subtype of indolent B cell non-Hodgkin lymphoma. The clinical course can be very heterogeneous with some patients being safely observed over many years without ever requiring treatment to other patients having more rapidly progressive disease requiring multiple lines of treatment for disease control. Front-line treatment of advanced FL has historically consisted of chemoimmunotherapy but has extended to immunomodulatory agents such as lenalidomide. In the relapsed setting, several exciting therapies that target the underlying biology and immune microenvironment have emerged, most notable among them include targeted therapies such as phosphoinositide-3 kinase and Enhancer of Zeste 2 Polycomb Repressive Complex 2 inhibitors and cellular therapies including chimeric antigen receptor T cells and bispecific T cell engagers. There are several combination therapies currently in clinical trials that appear promising. These therapies will likely reshape the treatment approach for patients with relapsed and refractory FL in the coming years. In this article, we provide a comprehensive review of the emerging and investigational therapies in FL and discuss how these agents will impact the therapeutic landscape in FL.

## Background

Follicular lymphoma (FL) is the second most common subtype of non-Hodgkin lymphoma (NHL) accounting for around 20% of all cases of NHL [1, 2]. FL most commonly presents either as a clinically enlarged lymph node(s) or incidentally on imaging performed for other reasons. The vast majority (80%) of cases do not have B-type symptoms or cytopenias at the time of diagnosis [3]. Although not strictly pathognomonic, 85–90% have a t(14:18) chromosomal translocation juxtaposing B cell lymphoma 2 (BCL2) to the immunoglobulin heavy chain promoter resulting in the escape of the transformed lymphocyte clone from the apoptotic death experienced by normal B-lymphocytes at the end of their lifespan. Interestingly, the t(14:18) can be found in nearly a quarter of healthy individuals regardless of age and gender with a similar distribution of breakpoints as in cases of FL [4, 5].

The extent of disease at diagnosis is the most important factor in the management of FL. Given the indolent nature of the disease, a majority of patients have advanced disease at diagnosis [6]. However, a subset of patients with well-localized disease limited to one to several lymph nodes can be successfully irradiated with long-term durable remissions. In this review, we provide a comprehensive overview of the emerging therapies and investigational approaches in relapsed/refractory (rel/ref) FL and briefly outline the advances in the frontline setting in advanced-stage disease requiring treatment.

## Follicular lymphoma biology and disease pathogenesis

It is now clear based on genomic studies that multiple other mutations are required in FL, including genes involved in chromatin remodeling, nuclear factor-kappa light chain enhancer of activated B cells (NF-κB) signaling, Janus Kinase-Signal transducer and activator of transcription proteins (Jak-Stat) signaling, and B cell development [7]. The chromatin remodeling mutations not only involve histone-modifying genes such as cAMP response element-binding protein (CREBBP), E1A binding protein 300 (EP300), Enhancer of zeste homolog 2 (EZH2), and Myocyte enhancer factor 2B (MEF2B) but also the histones themselves, particularly histone H1, resulting in interruption of histone-DNA interface and chromatin compaction, thus underlining the central importance of chromatin remodeling disruption in the pathogenesis of FL. These studies also support the existence of a founding common progenitor clone (CPC) rich in chromatin remodeling mutations that remain persistent despite therapy giving rise to now only FL cells but transformed FL in the majority of cases.

The lymphoma microenvironment also plays an important role in the pathogenesis of FL and has been extensively reviewed elsewhere [8]. A large seminal study of the gene expression signatures from untreated FL patients (*n* = 191) identified that non-malignant immune infiltrating cells had a significant impact on the prognosis [9]. Although past studies have investigated the prognostic significance or time to transformation of several different immune cell subtypes, including tumor-associated macrophages, Treg cells, and follicular dendritic cells [10–13]. A recent comprehensive study using cytometry time of flight (CyToF) on untreated FL samples (*n* = 31) showed a high degree of complexity of the infiltrating non-malignant T cells, distinguishing 12 distinct CD4 + T cell subsets, some of which had prognostic significance including a population lacking the co-stimulatory markers CD27 and CD28[14]. It will be exciting to evaluate the significance of these immune subpopulations in light of many of the newer approved and emerging FL therapies discussed in this review, many of which rely on immune-based mechanisms for their cytotoxicity. However, at present, enumeration and transcriptional profiling of lymphoma-associated immune cells remain investigational without a clear role in FL therapeutic decision making.

## Relapsed/refractory follicular lymphoma-approved and investigational approaches

**Fig. 1:**
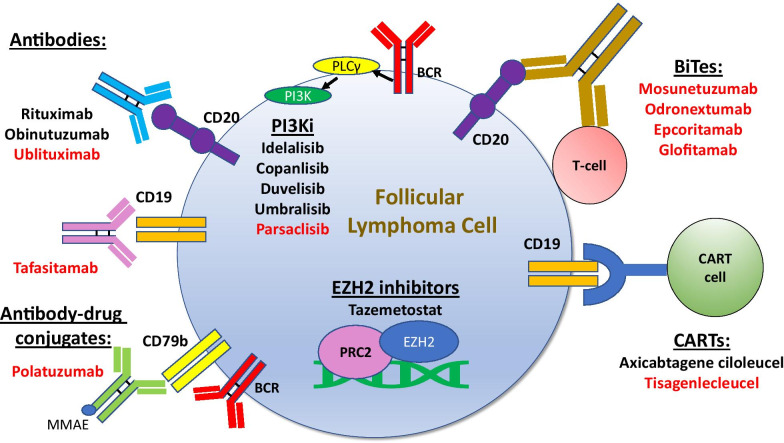
Approved (black) and emerging (red) therapies for follicular lymphoma

Previously, the only therapy available after the failure of frontline chemoimmunotherapy was either chemoimmunotherapy regimens or immunomodulatory (IMid) therapy. However, over the past few years, there have been significant advancements with new therapies approved for patients with rel/ref disease as well as several others on the horizon (Fig. 1). In this section, we discuss the most current data surrounding the use of several small molecule inhibitors in relapsed FL, including lenalidomide, phosphoinositide 3 kinase (PI3K) inhibitors, epigenetic therapies, as well as other inhibitors not currently approved but under investigation either alone or in combination with other approved agents. We then discuss the role of antibodies/antibody–drug conjugates, checkpoint inhibitors, and cellular therapies in rel/ref FL.

### Immunomodulators

Lenalidomide is a second-generation immunomodulatory agent that has a diverse range of anti-lymphoma activity which has been reviewed elsewhere [15]. It induces direct lymphoma cytotoxicity by promoting degradation of Ikaros zinc finger1 and Ikaros zinc finger3 (IKZF1 and IKZF3) by direct interaction and binding to the E3 ligase cereblon [16–18]. Degradation of cereblon also leads to an increase in p21 levels and a decrease in the levels of the transcription factor interferon regulatory factor 4 (IRF4) leading to inhibition of proliferation [19, 20]. In addition, lenalidomide has significant immune-mediated anti-lymphoma activity by proliferation and activation of natural killer (NK) cells resulting in increased immune synapse formation and antibody-dependent cell cytotoxicity [21]. Besides NK cells, lenalidomide leads to T cell stimulation and cytotoxicity and enhanced dendritic cell presentation [22, 23].

The activity of lenalidomide in rel/ref FL was initially studied in the CALGB 50,401 study, which was a randomized phase II three-arm study in which lenalidomide, rituximab, or lenalidomide plus rituximab (R2) were compared [24]. The rituximab-only arm was eventually dropped due to poor accrual. R2 was found to have a longer median progression-free survival (PFS) compared to lenalidomide alone (2 years vs 1.1 years). More patients in the R2 arm completed the full 12 months of treatment (63% vs 36%) due to more progression seen in the lenalidomide group. The R2 regimen was further studied in the phase III AUGMENT study which enrolled patients with FL or marginal zone lymphoma (MZL) who had previous chemotherapy or immunotherapy with at least 2 or more doses of prior rituximab. Patients with rituximab refractoriness were excluded in this trial [25]. Rituximab alone or R2 were compared with lenalidomide administered at 20 mg daily on days 1–21 of a 28-day cycle for 12 months while rituximab was given for the first 6 cycles. The median PFS was superior for patients receiving R2 compared to rituximab alone (39.4 months versus 14.1 months) but with a higher incidence of infections (63% v 49%), neutropenia (58% v 23%), and rash (32% v 12%). These data lead to subsequent FDA approval of R2 for rel/ref FL in May of 2019. The phase IIIb MAGNIFY trial is investigating if a longer duration of lenalidomide past one year would further improve patient outcomes. Patients in this trial are randomized to either rituximab maintenance or continued R2 after received one year of R2. An interim analysis at ASCO 2019 showed a tolerable safety profile with 38% of target enrollment randomized to maintenance accrued [26].

Lenalidomide has also be studied with obinutuzumab (LG), a glycoengineered anti-CD20 antibody with higher antibody-dependent cell cytotoxicity at the expense of diminished complement-dependent cytotoxicity [27]. This combination was demonstrated to be safe with lenalidomide dosing of 20 mg (12 cycles) and obinutuzumab dosage of 1000 mg/m2 (6 cycles) in a phase 1/2 study of relapsed indolent lymphomas [28]. In the GALEN study (*n* = 86), the overall response rate (ORR) and complete response (CR) rates were 79% and 38%, respectively [29]. It is important to note that 23% of patients included in the study were refractory to rituximab and 27% had progression within 24 months of initial treatment (POD24). The PFS at 2 years was 65%, with 34% of patients experiencing a serious adverse event (SAE) including basal cell carcinoma (6%), febrile neutropenia (5%), and infusion-related reactions (4%). As there have not been any randomized studies directly comparing LG versus R2 in rel/ref FL, the advantage of LG over R2 in this setting, particularly in patients without rituximab refractoriness, is currently unclear.

### Small molecule inhibitors

### PI3K inhibitors

The phosphoinositide 3 kinases (PI3Ks) are a family of intracellular signal transduction kinases that phosphorylate the 3’ position of the inositol ring of phosphatidylinositol present in the lipid membrane [30]. This gives rise to phosphatidylinositol 3,4,5 triphosphate (PIP3) which binds to the pleckstrin homology domain located in a variety of kinases including Protein Kinase B (PKB or Akt) and phosphoinositide-dependent kinase1 (PDK1 leading to increases in cell metabolism, growth and cell division. The PI3Ks are divided into three classes (class I, II, III) based on their structure, regulation, and lipid substrates [31]. The class I kinases include PI3K α, β, γ, and δ. PI3Kα and PI3Kβ are expressed in all cells while PI3Kδ and PI3Kγ and expressed primarily in leukocytes. In B cells, PI3Kδ plays an important and nonredundant role in B cell receptor (BCR) signaling and B cell activation [32–34]. PI3Kγ has been shown to play an important role in innate immune migration to the tumor microenvironment and inhibition of PI3Kγ was sufficient to prevent this action [35]. The best strategy of PI3K isoform inhibition for the greatest therapeutic efficacy in lymphomas remains an open question. Four PI3K inhibitors are currently approved for FL (idelalisib, copanlisib, duvelisib, umbralisib) with many others are under active investigation (Table 1).

**Table 1:** PI3K inhibitors in FL (includes completed and ongoing trials)

Treatment	Target specificity	Publication/NCT#	Phase	Total *n* [FL]	Median lines of prior therapy	ORR%^a^ [CR%]	Median PFS (mos)	Grade ≥ 3 AEs (%)^b^	Approved
*Single agent studies*									
Idelalisib	δ	[36]	II	125 [72]	4	45 [3]	11	Neutropenia [27], LFT elevation [13], diarrhea [13], PNA [7]	+
Copanlisib	α/δ	[39, 40]	II	142 [104]	3	59 [20]	12.5	Hyperglycemia [40], Hypertension [24], Neutropenia [24]	+
Duvelisib	δ/γ	[41]	II	129 [83]	3	42 [1]	9.5	Neutropenia [25], anemia [15], diarrhea [15], thrombocytopenia [12], LFT rise [5], lipase rise [7], colitis [5], PNA [5],	+
Umbralisib	δ, CK1ε	[44]	II	208 [117]	2	45 [5]	10.6	Neutropenia [11], diarrhea [10], LFT rise [7]	+
Parsaclisib	δ	NCT03126019	II	NA^c^	NA^c^	NA^c^	NA^c^	NA^c^	–
Zandelisib	δ	NCT03768505	II	NA^c^	NA^c^	NA^c^	NA^c^	NA^c^	–
YY-20394	δ	NCT04370405	II	NA^c^	NA^c^	NA^c^	NA^c^	NA^c^	–
Tenalisib	δ/γ	NCT03711578	II	NA^c^	NA^c^	NA^c^	NA^c^	NA^c^	–
*Combination studies*									
Duvelisib + BR or R	δ/γ	[50]	I	46 [15]	2	Duvelisib + R: 62 [19], Duvelisib + BR: 58 [17]^d^	Duvelisib + R: 10.7, Duvelisib + BR: 5.3^d^	Neutropenia [41], rash [20], diarrhea [13]	–
Umbralisib + ublituximab	δ, CK1ε	[51]	I	75 [19]	3	44 [22]	NP	Neutropenia [28], PNA [8], diarrhea [8], abdominal pain [7], thrombocytopenia [5]	–
Copanlisib + BR or R-CHOP	α/δ	NCT03711578	III	NA^c^	NA^c^	NA^c^	NA^c^	NA^c^	–
Duvelisib + nivolumab	δ/γ	NCT03892044	I	NA^c^	NA^c^	NA^c^	NA^c^	NA^c^	–
Idelalisib + pembrolizumab	δ	NCT02332980	II	NA^c^	NA^c^	NA^c^	NA^c^	NA^c^	–
Duvelisib + acalabrutinib	δ/γ	NCT04836832	I/II	NA^c^	NA^c^	NA^c^	NA^c^	NA^c^	–
Umbralisib + pembrolizumab	δ, CK1ε	NCT03283137	I	NA^c^	NA^c^	NA^c^	NA^c^	NA^c^	–
Umbralisib + ublituximab + lenalidomide	δ, CK1ε	NCT04635683	I	NA^c^	NA^c^	NA^c^	NA^c^	NA^c^	–

Abbreviations: AEs—adverse events; BR—bendamustine and rituximab; CR—complete response rate; NP—not presented; ORR—overall response rate; PFS—progression-free survival; PNA—pneumonia; R-CHOP—rituximab, cyclophosphamide, doxorubicin, vincristine, and prednisone

^a^Response rates for FL subset

^**b**^Grade ≥ 3 seen in at least 5% of all patients regardless of lymphoma subtype

^c^Not available, clinical trials are either ongoing or underway

^**d**^Results are for NHL, FL specific results were not presented due to the small number of pts

#### Approved PI3Ki

Idelalisib, a PI3K δ selective inhibitor, was the first approved PI3K inhibitor (PI3Ki) for relapsed FL based on a phase II study (*n* = 125) of heavily pretreated patients (median prior lines of therapy = 4) with indolent NHL (58% of which were FL), showing a median PFS of 11 months [36]. Potentially life-threatening autoimmune grade 3–4 AEs including colitis, hepatitis, and pneumonitis can occur at any time during treatment [37] and must be immediately recognized as the patients may require a short course of systemic steroids after exclusion of infectious causes [38]. In addition, opportunistic infections including pneumocystis jiroveci pneumonia (PJP) and cytomegalovirus (CMV) reactivation can occur with regular monitoring for CMV indicated while on therapy. Copanlisib is a PI3Kα/δ inhibitor which shown a median PFS of 12.5 months (*n* = 142 pts, 73% with FL) in the phase II CHRONOS-1 study leading to its approval [39, 40]. Its overall safety profile is more favorable than idelalisib, although hyperglycemia and hypertension were seen at a higher rate with copanlisib which can be explained in part, at least for the hyperglycemia, by greater PI3Kα inhibition.

Duvelisib is a PI3K δ/γ inhibitor currently approved for FL treatment based on the DYNAMO trial which showed a PFS of 9.5 months (*n* = 129 pts, 64% with FL), with diarrhea (48.8%), nausea (29.5%), and neutropenia (28.7%) being the most common side effects [41]. It is important to note that a greater proportion of patients included in the study were refractory to prior chemo-immunotherapy (77%).

Umbralisib is a PI3Kδ selective inhibitor that also has activity against casein kinase (CK)1-epsilon proteins. The CK1 proteins are a family of proteins that are involved in various processes including DNA repair, mitotic checkpoint signaling, and the immune response [42]. In particular, CK1ε plays an important role in the translation of lymphoma oncogenes as well as the Wnt-β catenin pathway. Of note, in a mouse model of CLL, treatment with umbralisib led to less intestinal and liver inflammation compared with idelalisib and duvelisib and correlated with a higher number of peripheral Treg cells in umbralisib mice, suggesting there may be a protective effect of CK1ε inhibition from the autoimmune toxicities induced by PI3K inhibition [43]. In a study of rel/ref indolent lymphomas including FL, single-agent umbralisib (*n* = 117) showed an ORR and CR rate of 45.3% and a 5.1%, respectively with a median PFS of 10.6 months[44] leading to its accelerated approval for rel/ref FL after at least three prior lines of therapy in February of 2021.

#### Emerging PI3Ki

Given the ongoing serious risk of autoimmune toxicity and infectious complications with long-term PI3K inhibition, particularly with strong PI3Kδ blockade, there is significant interest in balancing the anti-tumor effects with these toxicities. A possible way of mitigating these risks is with intermittent or induction/maintenance dosing schemes instead of continuous dosing. However, there are no ongoing trials with approved PI3K inhibitors comparing alternate dosing schemes and current approaches have rather focused on developing new PI3K inhibitors with better efficacy and/or safety profiles.

Parsaclisib is a structurally distinct PI3Kδ selective inhibitor with an overall greater potent inhibition of PI3Kδ than the other currently approved PI3K inhibitors. Early results from a phase1/2 of parsaclisib with and without itacitinib, a selective JAK1 inhibitor, showed an ORR and CR rate of 63% and 13% in the monotherapy arm in FL patients (*n* = 5) with results awaited from an ongoing phase 2 study in rel/ref FL (NCT03126019) [45]. Other PI3Kδ inhibitors currently in phase 2 clinical trials include zandelisib (ME-401, a selective PI3Kδ inhibitor, NCT03768505) and YY-20394 (a selective PI3Kδ inhibitor, NCT04370405) and tenalisib (PI3K δ/γ inhibitor, NCT03711578). Many drugs with dual PI3K/mammalian target of rapamycin (mTOR) inhibition have also been developed but their utility in providing improved efficacy at the expense of toxicity given their safety profiles in clinical trials is unclear [46].

#### Combinatorial approaches incorporating PI3Ki

Combinatorial approaches with approved PI3K inhibitors are currently ongoing and have been nicely detailed in a recent review [47]. In the cooperative group trial, when idelalisib was combined with R2, there was significant hepatic toxicity with 2 of 7 enrolled patients passing away from toxicity, one from hepatic failure and another from complications related to colitis leading to premature closure of the study [48]. Given the overall better safety profile of copanlisib, duvelisib and umbralisib compared to idelalisib, these PI3Kis may be better suited to be incorporated into combination treatment strategies either with chemotherapy or immunomodulatory agents. The preliminary results of a phase III study incorporating copanlisib into chemoimmunotherapy with either R-CHOP or BR in the relapsed setting showed an ORR and CR rate of 90% and 50%, respectively for BR + copanlisib (*n* = 10) and 100% and 30%, respectively for R-CHOP + copanlisib [49]. Four patients within the BR + copanlisib group and five patients within the R-CHOP + copanlisib group required discontinuation of treatment due to AEs. Pneumonitis did occur in one patient but no AEs related to colitis or hepatic failure were reported. Likewise, duvelisib was also studied in combination with BR in patients with relapsed chronic lymphocytic leukemia (CLL) or NHL (*n* = 29 including both indolent and aggressive lymphomas, 15 of which had FL) in phase I study with a dose-expansion phase [50]. Thirty-seven percent of patients experienced an SAE with 19.6% of patients determined to be related to duvelisib, including cases of CMV colitis, CMV esophagitis, inflammatory colitis, acute lung injury, and generalized rash, with no cases of hepatitis. The limited numbers of FL patients in this study preclude efficacy analysis, but given the serious toxicities when combining duvelisib with bendamustine likely related to the significant lymphosuppression, the role of combination duvelisib + bendamustine versus reserving duvelisib monotherapy for a later line is unclear. In the FRESCO trial (NCT02605694) duvelisib was combined with either rituximab or R-CHOP for patients with progression within the first 24 months after initial therapy but was subsequently withdrawn by the sponsor.

Combination studies with umbralisib are currently under investigation. The combination of obinutuzumab and umbralisib is one of the three arms of the ongoing SWOG1608 phase II trial (see Approach to the POD24 patient). An initial phase I study combining umbralisib with the novel anti-CD20 antibody ublituximab (U2) in rel/ref B-NHL and CLL found this combination to be safe with no new safety signals compared to the umbralisib alone with an ORR and CR rate of 46% and 17%, respectively[51]. As discussed earlier, given the overall reduced potential for autoimmune toxicity of umbralisib compared to the other PI3K inhibitors, combination therapies of umbralisib with lenalidomide/ anti-CD20 combinations may be safer with strong anti-FL activity. A clinical trial combining U2 along with lenalidomide (NCT04635683) is currently ongoing.

Another potential area of interest is combining PI3Ki with Bruton’s tyrosine kinase (BTK) inhibitors given the synergy and potentially non-overlapping toxicity. Acalabrutinib was studied in combination with PI3Kδ inhibitor and was shown to be safe and tolerable in early phase clinical trials in rel/ref B cell malignancies [52]. A phase Ib/II study is underway looking at the combination of acalabrutinib and duvelisib in rel/ref indolent NHLs (NCT04836832).

### Epigenetic therapies

Epigenetic regulation of lymphoid malignancies is currently an exciting area of research with significant potential for new therapies [53]. Given the shifts in methylated genes during transit through the germinal center (GC) during normal B cell differentiation, it is not surprising that epigenetic regulators have surfaced as undergoing mutation and dysregulation in germinal center (GC) derived B cell malignancies, such as FL [54]. Nearly 90% of FL have mutations in the histone methyltransferase, lysine methyltransferase 2A (KMT2A), and 60% in the acetyltransferase CREBBP. Around 80% of FL have co-occurring epigenetic mutations with most mutations being the loss of function mutations [55, 56]. In this section, we will review different classes of epigenetic modifiers that can serve as potential therapies in FL (Table 2).

**Table 2:** Epigenetic therapies in past and ongoing trials open to FL patients

Treatment	Target	Publication/NCT#	Phase	Total *n* [FL]	Median lines of prior therapy	ORR%^a^ [CR%]	Median PFS (mos)	Grade ≥ 3 AEs (%)^b^	Approved
*EZH2 inhibitors*									
Tazemetostat	EZH2	[60]	II	99 [99; MT 45, WT 45)	2	MT: 69 [13] WT: 35 [4]	MT: 13.8 WT: 11.1	None^c^	+
Tazemetostat + rituximab	EZH2	NCT04762160	II	NA^d^	NA^d^	NA^d^	NA^d^	NA^d^	–
Tazemetostat + lenalidomide + rituximab	EZH2	NCT04224493	I	NA^d^	NA^d^	NA^d^	NA^d^	NA^d^	–
*HDAC inhibitors*									
Vorinostat	Class 1 and 2 HDACs	[62]	II	50 [39]	1	49 [10]	20	Neutropenia [36], thrombocytopenia [23], lymphopenia [13], diarrhea [5], anorexia [7]	–
Vorinostat + rituximab	Class 1 and 2 HDACs	[64]	II	28 [22]	2	41 [27]	18.8	Lymphopenia [25], thrombocytopenia [18], neutropenia [11], fatigue [32], thrombosis [14], dehydration [11], hyperglycemia [11] hypotension [7], PNA [7],	–
Mocetinostat	HDAC 1,2,3,11	[66]	II	72 [31]	4	11 [4]	26.3	Fatigue [24], neutropenia [15], thrombocytopenia [12], anemia [8]	–
*DNMT inhibitors*									
5-azacytidine + R-CHOP	DNMT1	[68]	I	10 [3]	3	66 [33]	NP	Neutropenia [50], thrombocytopenia [40], anemia [20], abscess [10], anorexia [10], bacteremia [10], nausea [10]	–
*PRMT inhibitors*									
GSK3326595	PRMT5	NCT02783300^e^	I	NA^d^	NA^d^	NA^d^	NA^d^	NA^d^	–
JNJ-64619178	PRMT5	NCT03573310^e^	I	NA^d^	NA^d^	NA^d^	NA^d^	NA^d^	–
*BET inhibitors*									
CPI-0610	BRD2, BRD4	[74]	I	44 [8]	4	12 [0]	NP	NP	–

Abbreviations: AEs—adverse events; CR—complete response rate; MT—mutant EZH2; NP—not presented; ORR—overall response rate; PFS—progression-free survival; PNA—pneumonia; R-CHOP—rituximab, cyclophosphamide, doxorubicin, vincristine and prednisone; WT—wild type EZH2

^a^Response rates in FL subset

^**b**^Grade ≥ 3 seen in at least 5% of all patients regardless of lymphoma subtype

^**c**^No grade ≥ 3 events occurred in greater than 5% of patients, thrombocytopenia [3], neutropenia [3], anemia [2]

^d^Not available, clinical trials are ongoing

^e^first in human study enrolling patients with NHL or solid tumors

#### EZH2 inhibitors

EZH2 is histone lysine methyltransferase gene, which as a part of the polycomb repressor complex 2 (PRC2), catalyzes the addition of methyl groups to K27 of H3. This leads to transcriptional repression of target genes. EZH2 is important in the formation of GC in mice and it has been shown that mutations in EZH2 in addition to overexpression of BCL2 lead to B cell lymphomas [57]. EZH2 may also have immune modulatory effects wherein it suppresses the immune effector trafficking by repressing Th1 type cytokines [58]. EZH2 is mutated in ~ 20% of FL with the vast majority of mutations resulting in the substitution of tyrosine 641 leading to gain of function of methyltransferase activity. This distinguishes EZH2 mutations from most other epigenetic enzyme mutations which are loss of function mutations and thus provide a more readily available therapeutic target. Tazemetostat is a first-in-class oral inhibitor of EZH2 that has shown an overall favorable safety profile in the first in human phase I study of patients with rel/ref NHL [59]. The phase 2 study of tazemetostat in relapsed FL (who received 2 or more prior therapies, *n* = 99) demonstrated an ORR of 69% and 35% in EZH2 mutant (*n* = 45) and wild type (*n* = 54) patients, respectively, with a median PFS of 13.8 months and 11.1 months [60]. Grade 3 treatment-related AEs were infrequent and included thrombocytopenia (3%), neutropenia (3%), and anemia (2%). Tazemetostat is an attractive therapeutic option, especially in elderly patients or patients with co-morbidities that may preclude other therapies such as PI3Ki or chemotherapy given the clinical activity and favorable safety profile.

Valemetostat is a dual EZH1/2 inhibitor that has shown activity in both B and T cell NHLs in the Japanese population with an ORR of 53% with a particularly high response rate in T cell NHLs (ORR = 80%) [61]. The study is currently enrolling patients with T cell NHLs within the dose-expansion phase. Whether dual inhibition of EZH1/2 offers any benefit compared to selective EZH2 inhibition in FL is an open question that warrants further investigation.

#### Histone deacetylase inhibitors (HDACi)

Vorinostat is an oral inhibitor of class I and 2 HDACs and is currently approved for the treatment of T cell NHLs. As discussed previously, given the high rate of acetyltransferase loss of function mutations in FL, another potential therapeutic approach to counteract the loss of histone acetylation marks in these FLs would be to target the histone deacetylases to try to restore epigenetic homeostasis in these tumors. In a phase II study of relapsed indolent lymphomas and mantle cell lymphoma (MCL) (*n* = 56, 39 with FL), the ORR was 49% in the subset of patients with FL with a median PFS of 20 months. The toxicity was limited to cytopenias which were easily managed [62]. Mutation analysis revealed a mutation rate of 67.7% and 21.4% in CREBBP and EP300, respectively, with no correlation between the presence of a mutation and clinical response to vorinostat, although the limited number of patients precludes the determination of these mutations as predictive markers of response. Preclinical work has shown that inhibition of HDAC6 can lead to upregulation of CD20 with enhanced efficacy with anti-CD20 antibodies [63]. In a phase II study of rituximab in combination with vorinostat in newly diagnosed and rel/ref indolent B-NHLs (22 of 28 were FL), the ORR and median PFS were 41% and 18.8 months, respectively in the previously treated patients [64]. Patients who achieved a CR (*n* = 10) were allowed to come off treatment after 2 additional cycles of therapy with the option of retreatment upon relapse. Six remained in CR after a median follow-up of 27 months and of the 4 patients who underwent retreatment, 2 achieved a second CR, suggesting that time-limited therapy may be possible with this combination. It is unclear if vorinostat could resensitize rituximab refractory patients given the lack of information on rituximab refractoriness in the study. Studies of combination chemoimmunotherapy with vorinostat have largely been limited to diffuse large B cell lymphoma (DLBCL) with a few FL patients enrolled in these studies. The pan-HDAC inhibitor panobinostat that is currently approved for relapsed multiple myeloma has been studied in B-NHLs in early phase clinical trials in combination with mTOR inhibitor everolimus which inhibits cell proliferation by inhibiting protein translation [65]. Thrombocytopenia was the primary dose-limiting toxicity (DLT) with a median PFS of 4 months in FL patients. Studies on newer HDAC inhibitors, including abexinostat, mocetinostat, belinostat, entinostat, quisinostat, and chidamide have largely focused on T cell NHLs and aggressive lymphomas, except for mocetinostat which showed modest single-agent activity in FL. In a phase II trial of rel/ref DLBCL and FL, the ORR was 11.5% in the FL cohort (*n* = 31) [66].

#### Inhibitors of DNA methyltransferases (DNMT inhibitors)

The DNMTs have an opposing role to chromatin acetyltransferases by the methylation of DNA leading to transcriptional repression by maintaining or inducing a closed chromatin state thus precluding the binding of transcription factors. The DNMT inhibitors, 5-azacytidine, and decitabine have been established as effective therapeutics in myelodysplastic syndrome (MDS) and Acute Myeloid Leukemia (AML). In a small phase I study of low dose decitabine in patients with rel/ref CLL and DLBCL (*n* = 20), no responses were seen with 8 patients having a stable disease with correlative studies showing no differences in DNA methylation [67]. In another preliminary analysis of a study incorporating 5-azacytidine with R-CHOP in rel/ref lymphomas (*n* = 10, 3 with FL), one CR and one PR awere seen in the FL patients [68]. Given that only small DNMT inhibitor trials with limited numbers of FL patients have been reported, the potential of DNMT inhibitors in FL is currently unclear.

#### Inhibitors of Protein arginine methyltransferases (PRMT inhibitors)

The PRMTs catalyze the mono or dimethylation of histones, with PRMT5 being overexpressed in NHLs [69]. In addition, PRMT5, like EZH2, is required for GC formation and lymphoma survival through its interaction with BCL6, and thus, like EZH2, may also be important in the maintenance of epigenetic dysregulation in FL [70]. PRMT5 inhibitors have shown significant activity in pre-clinical models of Epstein Barr virus (EBV +) lymphomas, DLBCL, and MCL [71, 72]. Clinical trials of PRMT5 inhibitors including GSK3326595 (NCT02783300) and PF-06939999 (NCT03854227) are currently in the early stages of development. PRMT5 inhibition holds promise as another epigenetic therapy for FL.

#### Inhibitors of Bromodomain and Extraterminal Motif (BET) proteins

The BET proteins are a group of “histone readers” that act downstream of histone acetylation by binding to histone acetyl marks and recruiting transcription factors to DNA. Birabresib was the first BET to be evaluated in a clinical trial, but with no responses seen in DLBCL patients (*n* = 2) in the first-in-human study [73]. In another preliminary analysis of a dose escalation phase I study of the BET inhibitor CPI-0610 in B-NHL (*n* = 44, 8 with FL), one PR was seen in FL patients [74]. Thus far, response rates have been very modest and the high rate of histone acetyltransferase mutations in FL likely may result in resistance to this class of inhibitors. Restoration of acetyl marks by other epigenetic therapies may be required for the improvement of responses to these inhibitors.

#### Combinatorial approaches using epigenetic therapies

At present, the therapeutic potential of many epigenetic therapies may likely lie in novel combinations of already approved agents or rational combinations defined by synergy in the pre-clinical studies. A phase II trial of tazemetostat with rituximab (NCT04762160) and a phase I trial incorporating R2 with tazemetostat (NCT04224493) both in rel/ref FL after one prior line of therapy are currently underway. The interaction of other epigenetic modifiers with EZH2 is an exciting avenue of research. Preclinical work using a combination of acetyltransferase and methyltransferase inhibitors with EZH2 inhibition and profiling of epigenetic and downstream transcriptional changes will likely uncover interesting and novel combinations of epigenetic therapies with more potent clinical activity and will hopefully lead the way to rational clinical studies of epigenetic therapies in FL.

### BCL2 inhibitors

There was initially significant optimism in targeting the BCL2 in FL given the nearly universal presence of the 14:18 translocation in FL leading to overexpression of BCL2 resulting in escape from apoptosis. In contrast to other hematologic malignancies like CLL, MCL, AML, and MDS where venetoclax shows significant clinical activity, the response rate in FL has been modest with an ORR of 38% and a median PFS of 11 months [75]. This suggests the need to explore combination strategies with BCL2 inhibitors in the FL patient population.

The phase 2 CONTRALTO study was a three-arm study investigating the efficacy of venetoclax in combination with rituximab (chemo-free-arm, VR, Arm A), venetoclax with bendamustine and rituximab (VBR, Arm B), and standard of care bendamustine + rituximab (BR, Arm C) [76]. Venetoclax was given for a total of 12 cycles in both investigational arms with a safety run-in in the venetoclax + BR arm. Frequent dose reductions or delays were seen in Arm B due to neutropenia and diarrhea resulting in a reduction in the dose of BR. The ORR and CR rates were higher in the chemotherapy arms (Arm A 35% and 17%; Arm B 84% and 75%; Arm C 84% and 69%) with similar duration of response and PFS between Arm B and C. Due to the overlapping hematologic toxicities of venetoclax and chemotherapy, it is likely that a more optimized dosing schedule will need to be defined before combined chemotherapy and venetoclax will be useful in clinical practice.

The utility of BCL2 inhibition may be realized by combination with other targeted agents which may not only obviate the limited dosing due to hematologic toxicity but also increase efficacy based on more rationale mechanisms of cytotoxicity. Along these lines, the combination of ibrutinib and venetoclax is a therapy that is being explored across CLL, MCL as well as FL. Results of a phase I trial in rel/ref FL were recently presented at ASH 2020 and showed an ORR and CR rate (*n* = 16) of 69% and 25% with a safety profile similar to other studies of this combination with the most common grade ≥ 3 AEs including neutropenia (25%), thrombocytopenia (13%), lung infection (13%), and atrial fibrillation (6%) [77]. A phase II study of ibrutinib + venetoclax is currently ongoing (NCT02956382).

### BCR pathway inhibitors

Given the significant success of Btk inhibition in CLL and MCL, the efficacy of Btk inhibition in rel/ref FL was explored in a phase 2 trial of ibrutinib monotherapy (DAWN trial) [78]. Ibrutinib was given at a dosage of 560 mg until disease progression or unacceptable toxicity. At a median follow-up of 27.7 months (*n* = 110), the ORR and CR rates were 20.9% and 11% with a median PFS of 4.6 months and thus did not meet its primary efficacy endpoint. Another phase 2 trial showed an ORR and CR rate of 37.5% and 12.5% with a median PFS of 14 months [79]. The authors did note an increased response rate in rituximab sensitive disease (52.6% vs 16.7%) as well as resistance in patients with caspase recruitment domain family member11 (CARD11) mutations, suggesting pre-selection of patients based on these variables may allow better efficacy of Btki in rel/ref FL. As is the case with BCL2 inhibition, the role of Btk inhibition in FL is unclear at the present time and its potential will likely need to be defined with the use of rational combination treatments.

### Antibodies and antibody–drug conjugates (ADCs)

Ever since the introduction of rituximab that revolutionized the treatment of B cell NHL, new antibody therapies, including ofatumumab, obinutuzumab, and most recently tafasitamab and ADCs including brentuximab, and polatuzumab have been added into the lymphoma treatment armamentarium. Currently, only the anti-CD20 directed antibodies rituximab and obinutuzumab have approved indications for FL, while polatuzumab and tafasitamab have approvals in relapsed DLBCL in combination with bendamustine/rituximab and lenalidomide, respectively.

Polatuzumab is an anti-CD79 antibody conjugated to the cytotoxic molecule monomethyl auristatin E (MMAE) which was initially studied in combination with rituximab in both DLBCL and FL in the ROMULUS study [80]. In the FL cohort (25% of patients were refractory to rituximab) the ORR and CR rates were 70% and 45%, respectively with a median PFS of 15 months. However, 95% of the patients developed peripheral neuropathy during the course of the study, with a high rate at the 2.4 mg/kg dosing. Hence, a lower dose was recommended (1.8 mg/kg dosing) for future clinical trials. Polatutumab was studied in combination with BR versus BR alone in patients in rel/ref FL in the GO29365 trial [81]. Similar efficacy was seen with the ORR and CR rates of 77% and 69%, respectively in the BR + polatuzumab cohort and 73% and 63%, in the BR cohort with a median PFS of 17 months in each cohort. Polatuzumab has also been studied in combination with lenalidomide and obinutuzumab in phase Ib/II trial and demonstrated an ORR and CR rate of 76% and 65%, respectively [82]. Of note, 71% of pts who were refractory to their prior treatment achieved a CR, demonstrating the high activity of this combination in rel/ref FL.

Tafasitamab is a humanized antibody containing a hybrid IgG1/2 Fc domain directed against CD19 with enhanced antibody-dependent cellular cytotoxicity and antibody-dependent phagocytosis. Tafasitamab is currently approved for the treatment of rel/ref DLBCL in combination with lenalidomide based on the results of the L-MIND trial which demonstrated an ORR and CR rate of 43% and 18% with the most frequent grade 3 or higher treatment-emergent AEs being neutropenia (48%), thrombocytopenia (17%) and febrile neutropenia (12%)[83]. A phase III placebo-controlled study (lnMIND) of tafasitamab in combination with rituximab and lenalidomide is currently recruiting patients with rel/ref FL and MZL with an anticipated primary completion date of June of 2023(NCT04680052).

### Checkpoint blockade

Checkpoint blockade has shown mixed results in FL patients. Initial preclinical studies demonstrated that PD-1 expression is significantly upregulated on the surface of the peripheral blood and intratumoral CD4 + and CD8 + T cells in FL patients and PD-1 blockade resulted in significantly enhanced T cell function, thus confirming the immunomodulatory role of the PD-1/PDL-1 axis in FL-associated T cells [84]. A phase II study evaluated the activity of the combination of the PD-1 blocking antibody pembrolizumab with rituximab in patients after one or more prior lines of therapy who were not rituximab refractory [85]. Pembrolizumab was given at 200 mg every 3 weeks for up to 16 cycles while rituximab was given weekly for the first cycle of treatment. Of 25 patients evaluable for efficacy, the ORR and CR rates were 64% and 48%, respectively, although it should be noted that only 50% of enrolled patients had a lymphoma burden that met the GELF criteria. All immune-related AEs were either grade 1 or 2, but five patients discontinued therapy because of recurrent immune-related AEs. Baseline tumor PDL1 levels were not associated with response. The phase II Checkmate 140 study investigating nivolumab monotherapy (3 mg/kg every 2 weeks) in rel/ref FL showed an ORR of only 4% with median PFS of 2.2 months [86]. These results suggest that combination strategies with other immune stimulatory therapies will likely be required to realize the potential benefit of checkpoint blockade in rel/ref FL.

Despite the increased responses of combined PD-1/CTLA4 blockade in certain malignancies, a phase1b study of combining nivolumab and ipilimumab in rel/ref lymphoma and myeloma (*n* = 65 total, 5 with FL) showed an ORR and CR rate of 19% and 6%, respectively, in the B-NHL patients (*n* = 16) with only one response in the FL patients[87]. As expected, there was higher toxicity compared to past experience with nivolumab monotherapy. These results show that combined PD-1/CTLA4 is unlikely to have a role in FL immunotherapy.

Given the role of PI3K inhibition in promoting a more favorable immune microenvironment by a combination of immunomodulatory effects including depletion of Treg cells and improved macrophage inflammatory M1 to immunosuppressive M2 ratio [88, 89], another area of active interest is combining PI3Ki with immune checkpoint blockade. A phase I study with copanlisib with either nivolumab, a PD-1 blocking antibody, or with both nivolumab and ipilimumab, an antibody that blocks the inhibitory protein CTLA-4 on T cells in various cancers (NCT03502733) was initiated but subsequently suspended due to toxicity. A phase I study combining duvelisib and nivolumab (NCT03892044) which includes patients with transformed FL and CLL is ongoing as well as another study is investigating pembrolizumab either alone or with idelalisib or ibrutinib in patients with rel/ref low-grade B-NHL or CLL (NCT02332980). Given the reduced autoimmune toxicity seen with umbralisib relative to the other PI3K inhibitors, combining umbralisib with immune checkpoint blockade may provide a more favorable toxicity profile while still potentiating an immune-mediated anti-lymphoma response. Umbralisib is currently being combined with pembrolizumab in rel/ref B-NHL and CLL (NCT03283137).

Other clinical trials investigating combination strategies combining immune checkpoint blockade with rituximab and obinutuzumab (NCT03401853), rituximab, and lenalidomide (NCT02446457), ibrutinib (NCT02329847), and the bispecific anti-CD20 antibody mosunetuzumab (see cellular therapy section, NCT02500407) are currently ongoing.

CD47 is a surface protein expressed on nearly all cancers including lymphomas that provides an anti-phagocytic signal to enable cancer cells to evade macrophage-mediated killing [90]. CD47 antibodies not only induce apoptosis but also produce T cell responses by enhancing macrophage antigen presentation. Magrolimab (Hu5F9-G4) is a CD47 blocking antibody that is currently being studied in combination with rituximab in a phase Ib/2 trial of FL and DLBCL. Preliminary results from the phase Ib portion showed an ORR and CR rate of 71% and 43% in the FL cohort [91]. The treatment was well tolerated with the most common side effects being chills (41%), headache (41%), anemia (41%), and infusion-related reactions (36%). The phase II portion of the study is currently recruiting (NCT02953509). A phase I study of magrolimab in combination with obinutuzumab and venetoclax in indolent lymphomas is currently recruiting patients as well (NCT04599634). Other CD47 blocking antibodies currently in clinical trials enrolling patients with lymphoma include TTI-622 (NCT03530683) and ALX148 (NCT03013218).

### Cellular therapies

#### Chimeric antigen receptor T cell (CART) therapy

CD19 CART therapy has revolutionized the management of relapsed lymphoid malignancies offering potentially curative therapy to patients who were previously deemed incurable by contemporary therapies. Axicabtagene ciloleucel (axi-cel, Yescarta) received FDA approval in 2017 for rel/ref DLBCL after two prior lines of treatment based on the ZUMA-1 trial that showed an ORR and CR rate of 83% and 58% with a median PFS of 5.9 months [92]. Similarly, tisagenlecleucel (tisa-cel, Kymriah) received approval in 2018 based on results of the JULIET trial showing an ORR and CR rate of 52% and 40% with a relapse-free survival of 65% at 12 months. Both therapies have since been investigated in FL with impressive results.

##### Axicabtagene ciloleucel

The ZUMA-5 trial investigating axi-cel in indolent lymphomas enrolled patients with either FL or MZL after failing at least 2 prior lines of therapy (including an anti-CD20 therapy and an alkylating agent) with preliminary results recently reported at the 2020 ASH annual conference [93]. The study enrolled 146 patients, 124 of whom had rel/ref FL, many with high-risk disease including 55% of patients with progression of disease within 24 months of initial therapy (POD24) and 68% of who were refractory to their prior treatment. Axi-cel showed an ORR and CR rate of 94% and 60%, respectively in the FL patients with comparable responses across key risk groups. At data cutoff, 64% of FL patients had ongoing responses. Grade ≥ 3 cytokine release syndrome (CRS) and neurologic events only occurred in 6% and 15% of FL patients with one CRS-related death. In March of 2021, the FDA granted accelerated approval for axi-cel for patients with rel/ref FL after two or more prior lines of therapy.

##### Tisagenlecleucel

The ELARA study investigated tis-cel in patients with FL who received 2 prior lines of therapy, with the previous relapse within 6 months from the prior therapy or relapse after autologous hematopoietic cell transplant (auto-HCT) being other required criteria for enrollment [94]. Patients in this trial were also high-risk with 60% of patients with POD24 and 77% who were refractory to their last treatment. The ORR and CR rates were 82.7% and 65.4% in the intent to treat population with comparable responses across all prognostic groups. At a median follow-up of 9.9 months, median PFS had not been reached. There were no grade ≥ 3 CRS and only 2% had grade ≥ 3 neurologic events, all of which recovered which was consistent with the overall lower rate of CRS events in tisa-cel compared to axi-cel based on prior clinical experience. FDA approval has not yet been granted for tisa-cel at the time of this writing. Long-term follow-up is needed to evaluate the durability of response with CART therapy in rel/ref FL especially high-risk disease groups.

#### Bispecific T cell engager (BiTe) antibody therapy

BiTes are antibodies composed of two distinct antibody chains, one which recognizes an epitope present on T cells and the other of which is directed to an epitope on the target cell of interest resulting in direct cell-mediated toxicity by the cross-linked T cell to the tumor cell [95]. Although BiTes are not currently approved for FL at that time of this writing, the preliminary results with these agents in highly pre-treated FL appear promising in early phase clinical trials (Table 3). Larger efficacy studies are currently ongoing.

**Table 3:** BiTes currently in FL trials

BiTe	NCT#/publication	Route/Administration schedule	Phase	Total *n* [FL]	Median lines of prior therapy	% POD24	ORR%^a^ [CR%]	mPFS	CRS % [G ≥ 3]	Neuro % [G ≥ 3]	Approved
Mosunetuzumab	[96]	IV Qweekly for cycle 1, Q21 days for cycles ≥ 2, stopped after cycle 8 for CR	1	62 [62]	3	48	68 [50]	11.8	2	0	–
Odronextumab	[97]	IV Qweekly for weeks 1–12, Q2 weeks for weeks 12–36	1	96 [25]	3	NP	93 [71]	NP	7	3	–
Odronextumab	NCT03888105 [98]	IV Qweekly for weeks 1–12, Q2 weeks for weeks 12–36	II	NA^b^	NA^b^	NA^b^	NA^b^	NA^b^	NA^b^	NA^b^	–
Epcoritamab	[100]	sq Qweekly C1-2, Q2 weeks C3-6, Q4 weeks thereafter, 28 day cycles	I	67 [12]	3	NP	100 [25]	NP	0	3	–
Glofitamab	[108]	Obinutuzumab on D-7, weekly for two weeks then Q2 weeks for 28 weeks	I	171 [44]	3	NP	62 [52]^c^	11.8	3.5	1.2	–

Abbreviations: CRS—cytokine release syndrome; CR—complete response rate; Neuro—neurotoxicity; NP—not presented; ORR—overall response rate; PFS—progression-free survival; POD24—progression of disease within 24 months following chemoimmunotherapy; sq—subcutaneous; Qweekly—every week; Q2 weeks—every 2 weeks

^a^Response rates in FL subset

^b^Not available, clinical trial is ongoing

^c^for cohorts receiving ≥ 10 mg dosing

##### Mosunetuzumab

Mosunetuzumab is a CD20 directed BiTe that has been investigated in DLBCL and FL with updated results recently reported from a phase I dose escalation study in FL [96]. After weekly step-up administration during the first cycle, infusions were continued every 21 days for 8 cycles in patients who achieve CR or was continued for up to 17 cycles for patients who had stable or partial responses. The median number of prior treatments was 3 with 48% of patients with POD24 and 6% of patients who had prior CART therapy. The ORR and CR rate was 68% and 50%, respectively. At a median follow-up of 14.4 months, 62% of patients remained in remission with a median PFS of 11.8 months. SAEs occurred in 35% of patients, but only 21% of patients had CRS (one with grade ≥ 3) and 45% had neurologic AE (none with grade ≥ 3). The FDA has granted breakthrough therapy designation for mosunetuzumab in FL after 2 prior lines of therapy.

##### Odronextamab

Odronextamab (REGN1979), a CD20/CD3 BiTe, is an IgG4 antibody that is modified to reduce binding to the Fc receptor which has been studied in rel/ref B cell NHL [97]. Odronextamab was given every week for a total of 12 weeks followed by biweekly dosing for 12 more doses. Ninety-six patients were enrolled (25 with FL), 12 patients with prior CART. The CRS rate was 57% (*n* = 7 with grade ≥ 3). Grade 3 or higher neurotoxicity occurred in two patients [97]. The trial was suspended temporarily due to a patient's death from TLS for a protocol amendment. Responses were evaluated over a broad range of dosages with dosage-dependent responses seen. With treatment ≥ 80 mg, the FL cohort demonstrated an ORR of 95.5% (CR rate = 77.3%) with ≥ 5 mg [97]. A global phase II study is currently enrolling 5 separate disease cohorts of rel/ref NHLs, one of which is rel/ref FL [98]. However, at the time of this writing, a temporary hold was placed on both clinical trials due to a higher than anticipated rate of grade ≥ 3 CRS with a protocol amendment awaited to mitigate this risk.

##### Epcoritamab

Epcoritamab (GEN3013), a CD20/CD3 BiTe, is an IgG1 antibody that is unique in that it is administered subcutaneously rather than IV [99]. In pre-clinical models, subcutaneous administration demonstrated similar bioavailability and B cell depletion as IV administration but with lower plasma cytokine levels and was hypothesized to result in less CRS but with the same responses in patients [99]. Updated results of a dose escalation trial of epcoritamab administered subcutaneously in the outpatient setting in various lymphoma subtypes (18% with FL) were presented at the 2020 ASH conference [100]. The ORR and CR rate for FL patients (*n* = 8) was 100% and 25%, respectively. There were no grade ≥ 3 CRS events and only 3% had grade ≥ 3 neurologic events.

##### Glofitamab

Glofitamab (RO7082859) is a BiTE with a novel structure with bivalency for CD20 on lymphoma cells and monovalency for CD3 on T cells. In preclinical models of DLBCL, glofitamab had improved tumor cell kill compared to other bispecific antibodies [101]. Based on preclinical studies, obinutuzumab was given seven days prior to glofitamab infusion in a phase I study of rel/ref NHLs (*n* = 171, ~ 26% were FL) for peripheral and tissue B cell depletion to reduce the severity of CRS. In addition, a Bayesian modified continuous reassessment method was used for dose escalation. Efficacy was found to be dosage dependent in all NHL histologies with a higher ORR and CR rate for patients receiving ≥ 10 mg of 63.3% and 36.8% (*n* = 98) versus 53.8% and 52.0% for all dosing cohorts (*n* = 171). At the recommended phase 2 dosing (RP2D) of the weekly ramp of 2.5/10/30 mg, the ORR and CR rate for all histologies was 71.4% and 64.3%. The response rates in the FL cohort were dose dependent with an ORR and CR rate of 69% and 58.6% for the ≥ 10 mg cohorts (*n* = 29) and 70.5% and 47.7% for all FL dosing cohorts (*n* = 44). Although there was a high overall rate of CRS (50.3%), the incidence of grade ≥ 3 CRS was only 3.5%. As expected, CRS increased with dose but decreased in later cycles. Neurotoxicity was overall uncommon (3.5%) with grade ≥ 3 being only 1.8%. Several combination trials with glofitamab are currently planned for rel/ref NHL, including in combination with R-CHOP (NCT03467373) and or with polatuzumab or atezolizumab (NCT03533283).

### Approach to the POD24 patient

Patients with FL who progress within 24 months after completing first-line chemoimmunotherapy are defined as POD24. POD24 occurs in approximately 1 out of every 5 patients receiving R-CHOP as their primary therapy and is prognostically significant in this setting with a 5 year OS of 50% compared to 90% for non-POD24 patients [102]. Although validation of the predictive value of this endpoint in the case of other primary therapies, such as R2, is still needed, it is likely that progression after these other therapies also represents a high-risk disease that requires special approaches to achieving more durable responses after second-line therapy. The optimal management of the POD24 is an area of active investigation.

Escalation of second-line therapy with a consolidative auto-HCT in the patient fit for transplant has been a common approach. Although there are currently no randomized clinical trials to support this, studies suggest survival benefit of this approach in certain circumstances. A retrospective study using data from the Center for the International Blood and Bone marrow Transplant Research (CIMBTR) database and National Lymphocare Study (NCLS) compared 175 patients undergoing auto-HCT to 174 patients who did not within 2 years of treatment failure. Although this study did not find a difference in OS between these two groups, there was an improvement in 5 year OS (73% vs 60%) in favor of auto-HCT when looking at the subset of patients within one year of front-line treatment failure. In another analysis of the outcomes of POD24 patients enrolled on two German low-grade lymphoma clinical trials (GSLG1996 GSLG2000), there was a 5 year OS benefit among patients who received auto-HCT consolidation after second-line cytoreduction versus patients who underwent successful cytoreduction but did not undergo auto-HCT. The continued survival benefit of auto-HCT in the age of newer treatment modalities, such as obinutuzumab, immunomodulators, and PI3K inhibitors, is unclear and will have to be addressed with future outcome studies with patients undergoing treatment in the current era.

A pressing question currently is whether there is an optimal second-line therapy to offer POD24 patients after first-line chemoimmunotherapy failure. This question is currently being addressed by the SWOG1608 phase II intergroup trial (NCT03269669) which randomizes patients who have progressed after first-line chemoimmunotherapy into one of three arms: obinutuzumab and umbralisib, obinutuzumab and lenalidomide, or obinutuzumab and chemoimmunotherapy (bendamustine for previous R-CHOP or R-CHOP for previous bendamustine). This trial is currently recruiting with an anticipated completion at the end of 2022.

## Investigational approaches in Newly Diagnosed Advanced Stage FL

The majority of patients who receive frontline therapy with either chemoimmunotherapy or R2 will have a relatively durable response to therapy with a median PFS of 69.5 months for BR and a 3 year PFS of 77% for R2 [103, 104]. Long-term follow-up of the phase III PRIMA study compared patients who received rituximab maintenance therapy after chemoimmunotherapy (with either R-CHOP, R-CVP, or R-FCM) vs patients who did not receive maintenance rituximab showed an impressive median PFS of 10.5 years vs 4.1 years, respectively [105]. Further improvement on these already favorable results will require significantly long follow-up if unselected patient populations are recruited in trials. Targeting higher risk populations for trial enrollment has been difficult given the smaller number of these high-risk patients and the need for more accurate biomarkers predicting these high-risk patients at the start of treatment. Many past and current investigations into optimizing frontline therapy have focused on either incorporating novel treatments into induction and/or maintenance therapy or delivering less therapy with the hope of achieving the same durable responses.

The GALLIUM study investigated the benefit of obinutuzumab versus rituximab in combination with chemotherapy and found an improved estimated 3 year PFS (80.0% vs 73.3%) although no improvement in OS with a slightly higher frequency of grade ≥ 3 AE (74.6% vs 67.8%). It is important to note that all patients on this trial went on to receive maintenance obinutuzumab or rituximab so it is unclear if this same benefit would be seen without the addition of maintenance.

A recent randomized phase II trial incorporating bortezomib into BR induction (BR-R) either with rituximab maintenance or lenalidomide plus rituximab maintenance failed to demonstrate any additional benefit of incorporating either of these treatments to standard BR induction/rituximab maintenance therapy [106]. An ongoing phase II trial is investigating the addition of obinutuzumab with lenalidomide in the frontline setting to take advantage of the NK cell activating potential of lenalidomide to increase the ADCC of obinutuzumab [107]. Preliminary results are encouraging with an ORR and CR rate of 94% and 87% with a 2 year PFS of 96%[107]. Several clinical trials are currently ongoing using novel agents with anti-CD20 monoclonal antibodies in the frontline setting such as umbralisib with either rituximab (NCT03919175) or ublituximab (NCT03828448, NCT04508647), and tazemetostat with R-CHOP (NCT02889523).

Risk-adapted approaches are currently ongoing to evaluate the possibility of either de-escalation or escalation of therapy based on end of treatment responses. An ongoing Italian FOLL12 study (NCT02063685) is addressing if the end of induction therapy response as assessed by PET or MRD status can be used to either escalate or de-escalate maintenance therapy to ultimately improve upon PFS compared to standard rituximab maintenance therapy. Patients in the standard therapy arm will go on to receive rituximab maintenance after chemoimmunotherapy while patients in the experimental arm will be randomized into three groups: A) MRD negative, PET negative patients will be observed, B) MRD positive and PET negative patients will continue with rituximab maintenance, C) MRD positive and PET-positive patients will undergo consolidative radioimmunotherapy with [90]Y ibritumumab tiuxetan and rituximab maintenance. This trial has completed accrual and awaiting data maturation. UK and Australian PET response adapted therapy trial (PETReA) will randomize patients based on their end of induction therapy PET response: patients with negative PET will be randomized either to observation or maintenance rituximab and patients with positive PET will be randomized to maintenance rituximab or maintenance rituximab plus lenalidomide. This trial was opened in May of 2018 with a recruitment goal of around 1000 patients.

## Future perspectives

Although the majority of patients with advanced FL who require treatment are expected to have relatively good long-term outcomes based on current treatment approaches, there is still around 20% of patients who have a persistent relapsing course with very difficult to treat disease. This subset of patients needs to be prioritized for experimental therapies and investigational approaches. The SWOG1608 will likely address the approach for POD24 patients in the second-line setting. However, these patients will eventually relapse. Conventionally, allogeneic HCT was the only curative option in these high-risk patients but with the advent of several newer therapies in the second and later line setting, including CART therapy and the BiTes, the sequential use of therapies and the decision of when to pursue cellular therapy has become much more complex. Another important question of whether auto-HCT will continue to play a significant role in patients with POD24 or will it be supplanted by CART in the near future needs to be answered. A randomized clinical trial comparing auto-HCT to axi-cel therapy in this patient population, analogous to the ongoing ZUMA-7 trial in DLBCL, is warranted. Likewise, given the CR rate with the early BiTE therapy trials in heavily treated FL patients, these treatments will also likely play a role in the POD24 patient as well.

## Conclusions

Despite a better understanding of the genetic, epigenetic, and immunological landscape of FL, biomarker-driven and personalized approaches have remained elusive in the front-line treatment of FL, with “one size fits all” chemoimmunotherapy still being the most common approach for front line advanced FL treatment. The efficacy of the EZH2 inhibitor tazemetostat in the EZH2 mutated patient in the relapsed patient was a significant step forward in biomarker-driven therapy in FL. It will be interesting to discover whether patients in this subgroup would be able to avoid chemoimmunotherapy in their upfront treatment of FL if tazemetostat has significant activity in this setting, especially if it could be given for a fixed duration. Similarly, other chemo-free regimens like umbralisib and ublituximab given for a fixed duration or in a response-driven fashion, as in NCT04508647, are attractive but these types of frontline studies will require several years of follow-up to demonstrate non-inferiority or superiority.

Although chemoimmunotherapy was the mainstay for the treatment of FL for a long time, the past decade has seen an exciting number of novel targeted and cellular therapies in FL. Current and future trials on the horizon will continue to identify innovative treatment approaches and will hopefully lead to better outcomes in the most difficult to treat patients with FL.

## Data Availability

Not applicable.

## Abbreviations

FL: Follicular Lymphoma
rel/ref: Relapsed/Refractory
NHL: Non-Hodgkin’s lymphoma
Auto-HCT: Autologous hematopoietic stem cell transplant
CAR: Chimeric Antigen Receptor
BiTe: Bispecific T cell engager
Btk: Bruton’s tyrosine kinase
ORR: Overall Response Rate
CR: Complete Response
PFS: Progression-Free Survival
OS: Overall Survival
R2: Lenalidomide (Revlimid) and rituximab
